# Enhancing the oxygen evolution reaction activity of CuCo based hydroxides with V_2_CT_*x*_ MXene†

**DOI:** 10.1039/d4ta02700k

**Published:** 2024-08-02

**Authors:** Bastian Schmiedecke, Bing Wu, Thorsten Schultz, Aline Alencar Emerenciano, Namrata Sharma, Danielle A. Douglas-Henry, Apostolos Koutsioukis, Mehmet Turan Görüryılmaz, Valeria Nicolosi, Tristan Petit, Norbert Koch, Zdenek Sofer, Michelle P. Browne

**Affiliations:** a Helmholtz Young Investigator Group Electrocatalysis: Synthesis to Devices, Helmholtz-Zentrum Berlin für Materialien und Energie GmbH Albert-Einstein-Str. 15 12489 Berlin Germany Michelle.browne@helmholtz-berlin.de; b Department of Inorganic Chemistry, University of Chemistry and Technology Prague, Technická 5 166 28 Prague 6 Czech Republic; c Helmholtz-Zentrum Berlin für Materialien und Energie GmbH Berlin 14109 Germany; d Institut für Physik & IRIS Adlershof, Humboldt-Universität zu Berlin Berlin 12489 Germany; e Young Investigator Group Nanoscale Solid–Liquid Interfaces, Helmholtz-Zentrum Berlin für Materialien und Energie GmbH Albert-Einstein-Str. 15 12489 Berlin Germany; f School of Chemistry, CRANN and AMBER Research Centres, Trinity College Dublin College Green Dublin D02 PN40 Ireland

## Abstract

The oxygen evolution reaction (OER) is a key reaction in the production of green hydrogen by water electrolysis. In alkaline media, the current state of the art catalysts used for the OER are based on non-noble metal oxides. However, despite their huge potential as OER catalysts, these materials exhibit various disadvantages including lack of stability and conductivity that hinder the wide-spread utilization of these materials in alkaline electrolyzer devices. This study highlights the innovative chemical functionalization of a mixed copper cobalt hydroxide with the V_2_CT_*x*_ MXene to enhance the OER efficiency, addressing the need for effective electrocatalytic interfaces for sustainable hydrogen production. The herein synthesized CuCo@V_2_CT_*x*_ electrocatalysts demonstrate remarkable activity, outperforming the pure CuCo catalysts for the OER and moreover show increased efficiency after 12 hours of continuous operation. This strategic integration improved the water oxidation performance of the pure oxide material by improving the composite's hydrophilicity, charge transfer properties and ability to hinder Cu leaching. The materials were characterized using an array of materials characterization techniques to help decipher both structure of the composite materials after synthesis and to elucidate the reasoning for the OER enhancement for the composites. This work demonstrates the significant potential of TMO-based nanomaterials combined with V_2_CT_*x*_ for advanced innovative electrocatalytic interfaces in energy conversion applications.

## Abstract 2

V_2_CT_*x*_ MXene improves the OER performances of CuCo catalysts. The MXene over time reduces the leaching rate of the Cu by itself being preferentially leached which also allows for the higher OER activity to be maintained.

## Graphical abstract

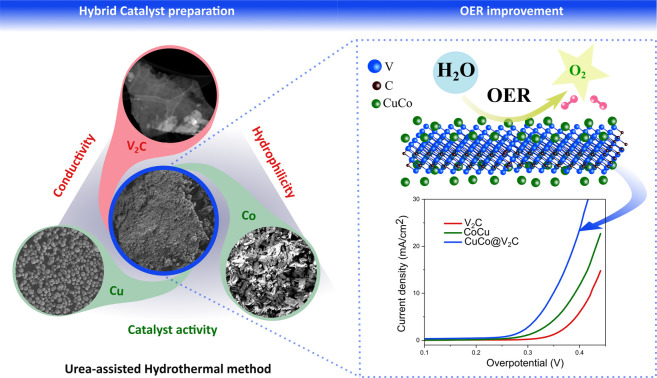

## 1. Introduction

Electrochemical water splitting, when combined with solar or wind technologies, is one potential route to make an alternative green fuel (*i.e.* green H_2_) to replace fossil derived fuels (*e.g.* oil, coal and gas) to power our buildings, vehicles or for feedstocks for important synthetic processes, such as the generation of ammonia through the Haber-Bosch process.^1^ During water splitting, H_2_ is produced at the cathode and is denoted as the hydrogen evolution reaction (HER). However, it is the anodic reaction, the oxygen evolution reaction (OER), which is the most energy intensive reaction during the water splitting process, as four electrons need to be transferred to generate one molecule of O_2_ while the cathodic reaction needs only two electrons to produce H_2_.^2^ Therefore, to make the overall process of water splitting more efficient, the OER needs to become less energy demanding.^1^

Currently, for the OER, the most studied catalysts in alkaline media, or for anion exchange membrane electrolyzers (AEMELs), are based on non-noble metals such as Ni and Co materials. However, these materials suffer from low conductivity and a loss in stability over time, which are essential prerequisites of an OER catalyst. There have been various studies to mix conductive aids with non-noble metal materials, however, the aids used are predominantly based on pure carbon materials such as graphene or carbon nanotubes.^3^ It has been shown that these carbon based materials somewhat corrode in alkaline environments (although not as corrosive as in acidic media) and can also contain metal impurities.^4,5^ Furthermore, the utilization of pure carbon supports has shown to decrease the faradaic efficiency of metal oxide materials for the OER and decrease the stability of the metal oxide compared to the same oxide on other supports.^6,7^ Recently, there have been reports emerging on utilising MXene materials as a conductive aid/support for OER catalysts.^8^

MXenes are a relatively new family of 2D materials, which are made up of transition metal carbides and nitrides, produced from MAX phases by various etching processes.^9^ A MAX phase has the general formula of M_*n*+1_AX_*n*_ where the M is an early transition metal, the A is an element from group 13 or 14 of the periodic table, and the X represents a carbon or nitrogen. During the etching process, carried out in a fluoride ion based solution, the A-element is removed from the MAX structure, causing the carbide layers to become terminated by OH^−^, O^−^, Cl^−^ or F^−^ groups, which are subsequently called ‘edge sites’.^10^ The resulting structure is known as ‘MXene’ with the most common/first synthesised MXene being Ti_3_C_2_T_*x*_.^9,10^ MXenes are known to be highly conductive, hydrophilic and mechanically durable due to their structures, which are essential properties for OER catalysts.^9^ However, to date MXenes are not known to contain active sites for the OER, as no MXenes with metals for promoting the OER have been successfully synthesised (*e.g.* Ni, Co).^11,12^

Furthermore, these edge sites on the MXenes are extremely important. The edge sites can be engineered/tailored to alter the materials inherent properties and therefore the conductivity, hydrophilicity, and the thermodynamic stability of the material is controllable. However, these edge sites can easily oxidise even in air, which results in the breakdown of the material into its respective oxide, *e.g.* Ti_3_C_2_T_*x*_ will oxidise into TiO_2_, which in turn leaves a material with reduced conductivity and hydrophilicity. Additionally and more importantly, it has been previously shown that Ti_3_C_2_T_*x*_ oxidises rapidly under OER conditions to TiO_2_ hence, this material alone is not an appropriate OER catalyst.^13^ However, there are many strategies being developed to block or ‘cap’ these edge sites to hinder or slow down the oxidation process.

One way to engineer the edge sites for the OER is to use active OER materials, first row inexpensive MO_*x*_, such as Ni, Fe, Co and Mn, and anchor them onto the MXene edge sites in order to hinder the oxidation process. There have been multiple reports on the manipulation of Ti_3_C_2_T_*x*_ with metal hydroxides/oxides which show an increase in the OER for the metal oxide/Ti_3_C_2_T_*x*_ composite.^13–17^ For example, Benchakar and co-workers have reported that a cobalt layered double hydroxide (Co-LDH)/Ti_3_C_2_T_*x*_ material synthesised by a solvothermal synthesis results in the promotion of the OER by 50 mV when compared to the pure Co-LDH catalysts in alkaline electrolyte.^14^ To date, there are only few reports on OER metal oxide catalysts with other MXene materials.^18,19^ In one recent study, Rogach *et al.* compare the OER activity of three different MXene supports (Ti_3_C_2_T_*x*_, V_2_CT_*x*_ and NbCT_*x*_) with Co single atoms made by an ice photo-reduction route.^19^ This study reveals that the V_2_CT_*x*_ with Co single atoms exhibits the lowest OER overpotentials, however, over a period of 10 hours, this composite results in a loss of activity. The authors postulate from experimental and theoretical studies that the superior OER capabilities of the V_2_CT_*x*_ are attributed to the re-distribution of the electronic structure of the Co atoms due to a higher rate of electron transfer.^19^ Furthermore, various studies have suggested that the OER performance of Co oxides can be improved by the addition of Cu into its structure in alkaline media.^20,21^ For example, Scott and co-workers demonstrated that CuCo oxides outperformed Co oxides in a three electrode cell and in an AEMEL device.^20^ Recently, Alishahi and team synthesised a CuCo and a CuCo/Ti_3_C_2_T_*x*_ (MXene) catalyst for alkaline OER, which exhibited overpotentials at 10 mA cm^−2^ of 470 and 380 mV, respectively.^22^ This study showed that the addition of the most common MXene, Ti_3_C_2_T_*x*_, is effective in improving the OER activity of a CuCo based material. To our knowledge, the effect of V_2_CT_*x*_, the material when combined with Co single atoms showed the best OER performance compared to the same Co single atoms on Ti_3_C_2_T_*x*_, on the OER properties of CuCo materials has not been investigated.^19^

In this work, a range of novel CuCo based V_2_CT_*x*_ composites are fabricated through a hydrothermal route and investigated for the OER. The V_2_CT_*x*_ amount varies in the composite materials to determine the effect of the MXene content on the OER. These materials are characterized by a suite of techniques to determine the surface and bulk chemical composition before and after the OER, which allows for changes in the chemical composition to be attributed for changes in the OER performance of pure CuCo and MXene composite materials.

## 2. Material and methods

### 2.1. Materials and chemicals

The materials and chemicals used in the current study were a graphite rod (redox.me), a mercury–mercury oxide (Hg/HgO) reference electrode (redox.me), a glassy carbon disc electrode (ALS instruments) and Nafion (Sigma Aldrich). Sodium hydroxide pellets (≥98%, reagent-grade), ethanol (≥99%, reagent-grade), isopropanol (≥99%, reagent-grade), Co(CH_3_COO)_2_·4H_2_O (≥99% metal basis, *M* of 249.08 g mol^−1^), Cu(CH_3_COO)_2_·H_2_O (≥99% metal basis, *M* of 199.65 g mol^−1^), V_2_AlC MAX phase (Jinzhou Haixin Metal Materials, China), NaF (Lachner), HCl (Lachner), TBAOH (Sigma-Aldrich) and urea (≥99%, ACS reagent, *M* of 60.06 g mol^−1^) were purchased from Sigma-Aldrich. For sample preparation and dilutions, ultrapure water with a resistivity of 18.2 MΩ cm was used.

### 2.2. Preparation of V_2_CT_*x*_

The exfoliated V_2_CT_*x*_ MXene was prepared by removing the Al layer from its MAX phase V_2_AlC using a mixed HCl + NaF etching solution. The detailed experimental process is as follows: 4.5 g of NaF was stirred and dissolved in 60 ml of distilled water, followed by the addition of 60 ml of concentrated hydrochloric acid. Next, 5 g of V_2_AlC MAX phase was slowly added to the above solution and stirred for 1 hour under an atmospheric environment until no obvious bubbles were generated. The mixture was then sealed in a stainless-steel autoclave with a Teflon liner. The mixture was continuously stirred in a 90 °C oil bath for 3 days. Due to the difficulty in completely removing Al from the MAX phase, the solid product from the first etching was subjected to the same etching steps again. The product was then centrifuged three times with distilled water to obtain an etched precursor. Subsequently, the precursor was stirred in 20 ml of 20% TBAOH solution for 24 hours for exfoliation, and then diluted to a 100 ml suspension and stirred for an additional 24 hours. The suspension was centrifuged at 10 000 rpm and washed three times with an ethanol/water solution in a 1 : 4 ratio to remove residual TBAOH. Finally, centrifugation at 1000 rpm was used to remove unetched MAX phase or other solid impurities. This process yielded well-exfoliated V_2_CT_*x*_ MXene for further use.

### 2.3. Preparation of CC1, CC10, CC25, CC50 and the pure Cu, Co and CuCo materials

CuCo@V_2_CT_*x*_ was synthesized through a urea-assisted hydrothermal method. In a typical synthesis process, V_2_CT_*x*_ was used from a colloidal solution (1.47 mg ml^−1^) and then, 5 mmol urea, 1 mmol Cu(CH_3_COO)_2_·H_2_O and 2 mmol Co(CH_3_COO)_2_·4H_2_O were added to the solution, and it was stirred for 30 minutes to fully dissolve all the compounds. The solution was transferred into a Teflon-lined stainless-steel autoclave and kept at 120 °C for 6 h for hydrothermal treatment. After cooling down to room temperature, the precipitate was collected by centrifugation at 5000 rpm for 10 min, and then repeatedly washed with deionized water (3×) and ethanol (3×). The sediment was dried at 60 °C for 10 h. The collected powder samples were labelled as CC1, CC10, CC25, and CC50, for 1, 10, 25, and 50 molar mass% V_2_CT_*x*_ content, respectively. The pure materials, labelled pure Co, pure Cu and pure CuCo, were synthesized using the same method without addition of V_2_CT_*x*_.

### 2.4. Electrochemical measurements

The electrochemical performance of the materials was characterized in a standard three-electrode cell in alkaline aqueous solution at RT. 1.0 M NaOH was used as electrolyte, prepared from NaOH pellets. A standard mercury–mercury oxide electrode (mercury/mercury oxide Hg/HgO) was employed as a reference electrode (RE) and a graphite rod as a counter electrode (CE). The working electrode was a 3 mm diameter glassy carbon (GC) disk with a geometric surface area of 0.0707 cm^2^. The cell was connected to an electrochemical workstation (PalmSens) and a rotating ring disc electrode (RRDE, ALS-Japan).

A catalytic ink was prepared for every material by dispersing 10 mg of catalytic powder in 1 ml DI water/isopropanol solution (1 : 1) and 8 μL Nafion. The solution was ultrasonicated for 10 min to form a homogenous ink. To reach a catalyst loading of 1.6 mg cm^−2^, 11.3 μL ink was deposited onto the polished GC disk and allowed to dry in air at RT. All measurements were performed in nitrogen saturated 1 mol L^−1^ NaOH electrolyte. Cyclic voltammetry (CV) profiles and linear sweep voltammetry (LSV) data were acquired between the potential ranges −0.2 and 0.6 V *vs.* RHE and 0.0 V and 0.85 V *vs.* RHE, respectively. First, each catalyst was subjected to 8 CV cycles in the potential range at a scan rate of 40 mV s^−1^. Polarization and Tafel plot measurements were performed at a scan rate of 1 mV s^−1^ and a rotation rate of 1600 rpm. To determine the resistance of the cell, electrochemical impedance spectroscopy (EIS) was recorded in a frequency range between 1 Hz and 1 mHz, with an oscillation amplitude of 10 mV in a non-faradaic region. To determine the charge transfer resistance of the materials under operation, EIS measurements were conducted in the OER region at 1.6 V *vs.* RHE. To test the long term stability of the catalyst, chronopotentiometry was executed at a constant current density of 10 mA cm^−2^ for various times.

## 3. Results & discussion

### 3.1. Structural characterization

**Fig. 1:**
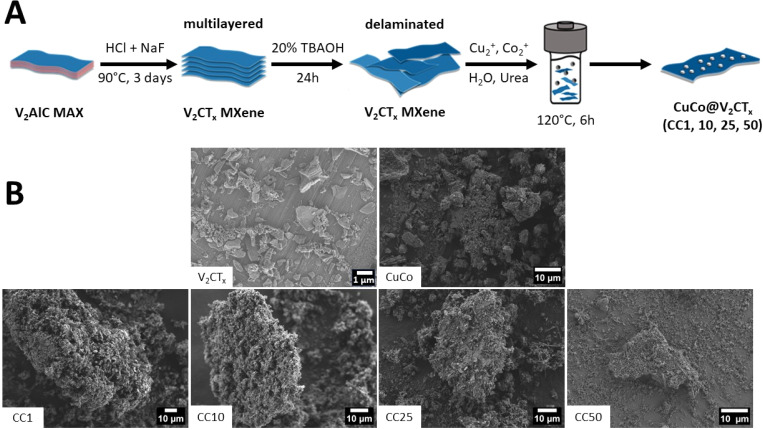
(A) Schematic of the synthesis procedure of the CuCo V_2_CT_*x*_ composites and (B) SEM images of V_2_CT_*X*_, CuCo, CC1, CC10, CC25 and CC50.

In this work, the targeted CuCo/V_2_CT_*x*_ composites were fabricated by a two-step urea assisted hydrothermal process, Fig. 1A. In the first step of the process, the V_2_CT_*x*_ (MXene) was produced by etching the V_2_AlC by *in situ* exfoliation with NaF and HCl to produce multi-layer V_2_CT_*x*_ which was then delaminated to achieve V_2_CT_*x*_. The composites were then prepared by adding the different wt% amounts of V_2_CT_*x*_ to an autoclave along with copper acetate, cobalt acetate, urea and distilled water. The reactant solutions were annealed in an oven at 120 °C for 6 hours (see experimental section for a more detailed description).

The concentration of V_2_CT_*x*_ in the composite materials were 1, 10, 25 and 50 wt% and from here on the resulting composite catalysts will be denoted as CC1, CC10, CC25 and CC50, respectively. Pure Co, pure Cu and CuCo catalysts were also synthesized under the same reaction conditions but without addition of V_2_CT_*x*_. Hence, in total eight catalysts were made in this work. To identify the chemical and structural properties of the prepared catalysts before the OER studies, the materials were characterized by SEM, XRD, and XPS.

SEM was carried out on the various catalysts to determine their morphological characteristics, Fig. 1B. The V_2_CT_*x*_ exhibits typical flake-like dimensions seen for delaminated MXenes, while the pure CuCo is composed of aggregated material. The CuCo/V_2_CT_*x*_ composite materials aggregated material is predominantly found around the flake-like structures from the V_2_CT_*x*_. The composite materials do not resemble the morphologies of the pure Co or Cu -based materials. The pure Co material exhibits a coarse leaf-like morphology, and the pure Cu material has a spherical shape morphology (Fig. S1†).

**Fig. 2:**
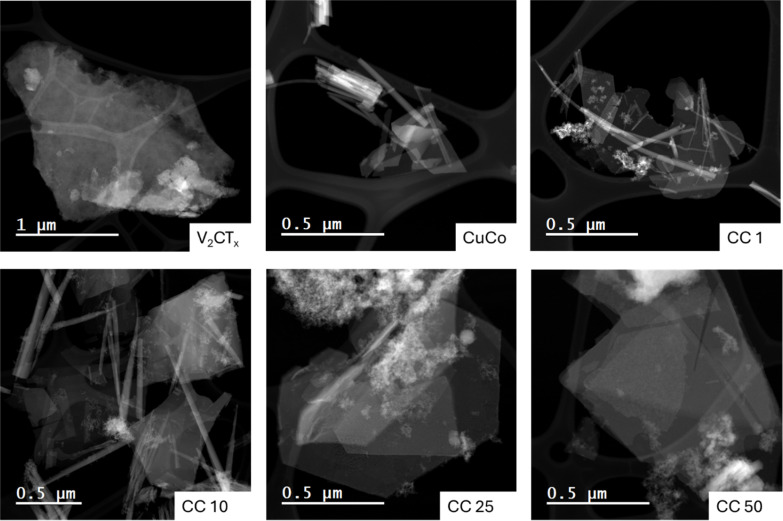
HAADF-STEM images of V_2_CT_*X*_, CuCo, CC1, CC10, CC25 and CC50.

To get a more in-depth account of the morphology and elemental composition of the pure and composite materials, scanning transmission electron microscopy (STEM) with energy dispersive X-ray spectroscopy (EDS) mapping was carried out, Fig. 2 and S2–S7.† The STEM image of the V_2_CT_*x*_ confirms the flake morphology observed with the SEM and the EDS map shows that the entire flake consists of vanadium, Fig. S2.† The CuCo STEM image, Fig. 2, shows that the aggregated material observed in the SEM consists of flake and rod-like structures. From the EDS maps of the CuCo sample, Fig. S3,† the Co and Cu are present in both structures. For the CC1 and CC10 materials, both contain flake and rod-like morphologies. The rod-like structures contain a good distribution of Cu, Co and V while the flakes have a higher distribution of Co when compared to Cu and V from the EDS mapping in Fig. S4 and S5.† From the STEM images in Fig. 2 of the CC25 and CC50, it is evident that a foam-like structure is present which is less prominent in the other composites. From the EDS maps in Fig. S6 and S7† for the CC25 and CC50 materials, this foam structure contains a higher concentration of V with the Cu and Co compared to the rod or flake structures present.

**Fig. 3:**
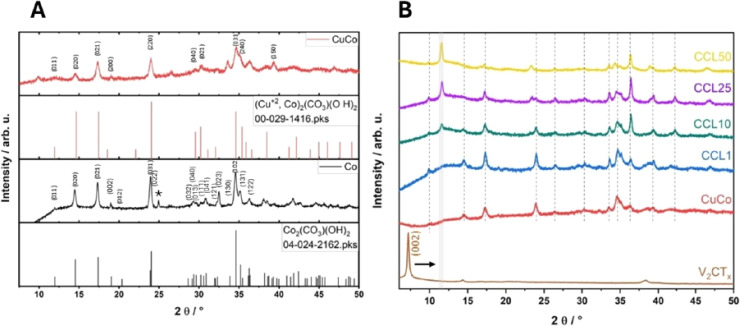
(A) XRD pattern of pure Co and pure CuCo (B) XRD pattern of V_2_CT_*X*_, CuCo, CC1, CC10, CC25 and CC50. (* = peak not in corresponding reference pattern).

X-Ray diffraction of the pure and composite catalysts was carried out to determine the crystal structure and to determine if the addition of the V_2_CT_*x*_ to the CuCo had any effects on the composites final structure, Fig. 3. The pure Co catalyst can be predominantly indexed to cobalt carbonate hydroxide (ICDD: 04-024-2126, Fig. 3A). The pure Co material exhibits major peaks at 14.5, 17.4, 24.0, 32.3 and 34.6° which corresponds to the (020), (021), (022), (023), (102) and (023) reflections of the Co_2_(CO_3_)(OH)_2_ while the peak at about 24.8° indicates the presence of another Co_2_(CO_3_)(OH)_2_ phase *i.e.* Co(CO_3_)_0.5_OH·0.11H_2_O.^23^ Furthermore, the major diffraction peaks at 14.8, 17.4, 24.0, 34.6, 35.4, 36.5 and 38.4° for the mixed CuCo material can be referenced to the (020), (120), (220), (031), (240), (330) and the (150) planes of the mineral Kolwezite (Cu^+2^, Co)_2_(CO_3_)(OH)_2_, Fig. 3A (ICDD: 00-029-1416). The additional peaks at 2Theta values of ∼10, 27 and 34° may again indicate the presence of Co(CO_3_)_0.5_OH·0.11H_2_O in the material.^23^

From Fig. 3B, in relation to the Kolwezite peaks in the composite materials, there are also some shifts in the 2theta positions and the intensity of the peaks which shows that the V_2_CT_*x*_ is influencing the Kolwezite crystal structure. For example, the Kolwezite (220) and (031) reflection at 24 and 35.4°, respectively, decreases with increasing V_2_CT_*x*_. Furthermore, the (220) reflection at 24° changes significantly upon subsequent V_2_CT_*x*_ additions for the materials CC10-50. For the CC10, this peak is lower in intensity compared to CC1, then the peak for the CC25 splits into two peaks and then for CC50, only the newer peak at the lower 2theta value is present.

Furthermore, the diffraction pattern of the pure V_2_CT_*x*_ shows that the V_2_AlC was fully etched and delaminated into the MXene counterpart from the presence of the strong (002) and the (004) reflections at 7.2° and 14.4°.^24,25^ The peak at 37.8° may be associated with LiF residue left over from the etching procedure.^24^ The XRD patterns of the CC1-50 are present in Fig. 3B and all show reflections associated with both Kolwezite and the V_2_CT_*x*_, however, some diffraction peaks are altered compared to the pure materials. The (002) peak of the V_2_CT_*x*_ in the composites (CC1-50) is shifted to a higher 2theta value of 11.5°, indicating a reduction in the interlayer spacing, possibly due to re-stacking of the single V_2_CT_*x*_ flakes upon drying.^26,27^ Additionally, the presence of vanadium oxide materials in the bulk of the composites can also be ruled out as there are no additional reflections associated to V_2_O_3_, V_2_O_5_ or V_3_O_8_ in the XRD patterns.^24^

**Fig. 4:**
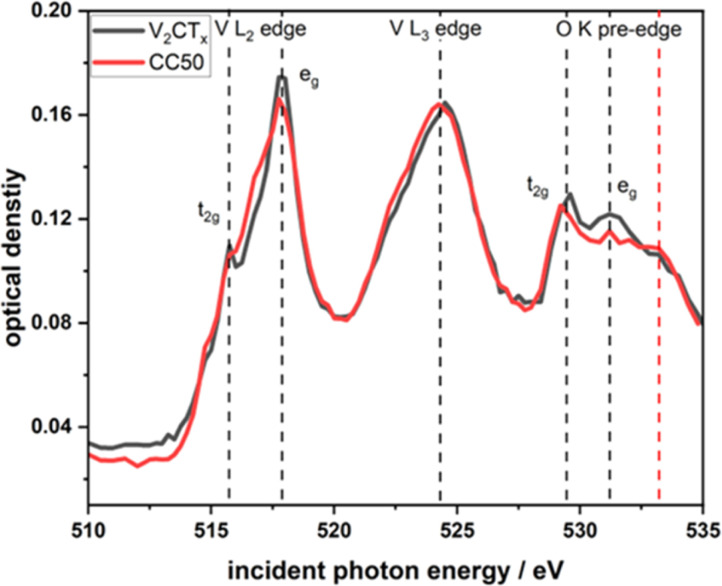
Comparative V–L edge and O–K pre-edge XANES spectra obtained from STXM of few-layered flakes of V_2_CT_*x*_ and CC50.

XANES at the V L edge and O K edge (Fig. 4) were acquired on single flakes of the pure V_2_CT_*x*_ and the CC50 using STXM (Fig. S8A and B†). STXM is based on transmission detection which is sensitive to the bulk of probed MXene flakes.^28^ For the pure V_2_CT_*x*_ and the CC50 materials, the V L-edge consists of 2 major peaks corresponding to L_3_ and L_2_-edge at 517.6 eV and 524 eV respectively. These peaks are associated with the electronic transitions from the V 2p_3/2_ and 2p_1/2_ core levels to the 3d orbital. Hence, crystal field splitting of 3d orbital gives rise to t_2g_ and e_g_ orbitals for both edges (Fig. 4). Fig. S2† shows the chemical map of V_2_CT_*x*_ and CC50 measured at 517 eV peak absorption energies for V L_3_ edge. A comparison of the V L_3_ edge t_2g_/e_g_ intensity ratio shows that there are no significant changes in V oxidation state between the pure V_2_CT_*x*_ (t_2g_/e_g_ intensity ratio: 1 : 0.60) and the CC50 (t_2g_/e_g_ intensity ratio: 1 : 0.62) material. This would indicate that the V_2_CT_*x*_ is not altered in the CC50 materials. For V_2_CT_*x*_ the O K pre-edge originates from electronic transitions from O 1s to O 2p orbital hybridized with V 3d orbitals. Overlap of oxygen 2p and vanadium 3d orbitals results in a set of bonding and anti-bonding orbitals *i.e.* t_2g_ and e_g_ orbitals. CC50 shows similar t_2g_ and e_g_ orbital splitting to pure V_2_CT_*x*_ along with an additional peak at 533.2 which could be due to the oxide layer formed by CuCo oxide over the MXene surface.

**Fig. 5:**
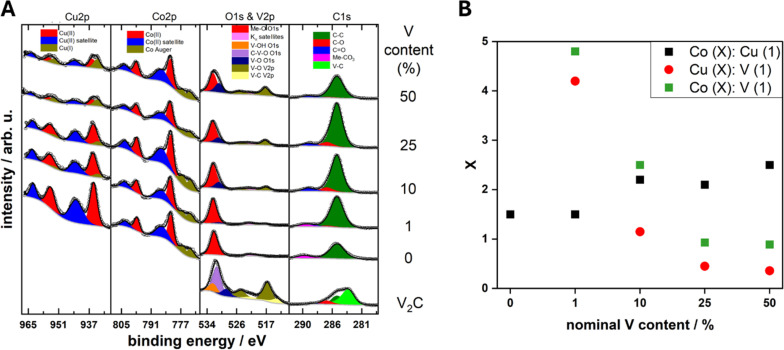
(A) XPS core levels of the V_2_CT_*x*_, CuCo, CC1, CC10, CC25 and CC50 and (B) estimated ratios of the surface Co : Cu, Cu : V and Co : V content from XPS core level areas.

XPS, Fig. 5, was utilized to evaluate the surface chemistry of the pure and composite materials to assess if it differs from the bulk of the materials probed by STXM. The XPS spectra are shown in Fig. 5A and B. In relation to the pure MXene sample, the presence of the V–C peak in the C1s region at 282 eV confirms that the V_2_CT_*x*_ structure is preserved after delamination. For the same sample, the V 2p region also confirms the presence of V^2+^ at a binding energy of about 513 eV, which is related to V–C bonds, and the presence of V^4+^ at 516 eV mostly associated to V–O bonds on the MXene surface.^29^ From the V 2p region, the state of the vanadium for the composites at the surface is in a V^4+^ state.^30,31^ No V^2+^ component is detected on the composite materials. This may be related to the short probing depth of XPS and the fact that the CuCo materials stand on top of the vanadium atoms. The surface of V_2_CT_*x*_ is highly susceptible to oxidation and oxygen species on the surface are very common.^32^ The O 1s peak for the pure MXene can be fitted to three peaks at 529, 531 and 532 eV which correspond to V–O, C–V–O and V–OH bonds. The V–O bonds can be attributed to surface vanadium oxides, the C–V–O is indicative of oxygen terminated V_2_CT_*x*_ while the V–OH may be present due to OH terminations on the V_2_CT_*x*_.^27^ The fact that no change in the vanadium oxidation state was observed from STXM, which is bulk-sensitive, confirms that the oxidation concerns mostly the top layer of the few-layered MXene flakes.

For the pure CuCo catalyst, the Co 2p and Cu 2p core levels correspond to Co^2+^ and Cu^2+^ due to the main peaks at 782 and 936 eV, respectively, and the characteristic satellite structure.^30,33^ Additionally in the C 1s region, the CuCo catalyst exhibits a metal-carbonate bond around 290 eV and a metal–oxygen peak in the O 1s region at around 532 eV, which further confirms the synthesis of a copper cobalt carbonate hydroxide. Interestingly, upon the addition of V_2_CT_*x*_ in the composite, a partial reduction of the Cu oxidation state from Cu^2+^ to Cu^1+^ is observed at the Cu 2p core level.^30^ Furthermore, the amount of Cu^1+^ increases with the V_2_CT_*x*_ concentration. Additionally, the ratio of Co : Cu at the surface of the composites increases with V_2_CT_*x*_ which effectively results in the enrichment of the surface with Co^2+^ in comparison to the Cu^2+/1+^ species from the XPS, Fig. 3C. The amount of vanadium species at the surface also increases progressively with the concentration of V_2_CT_*x*_ in the composite.

### 3.2. Electrochemical characterization

**Fig. 6:**
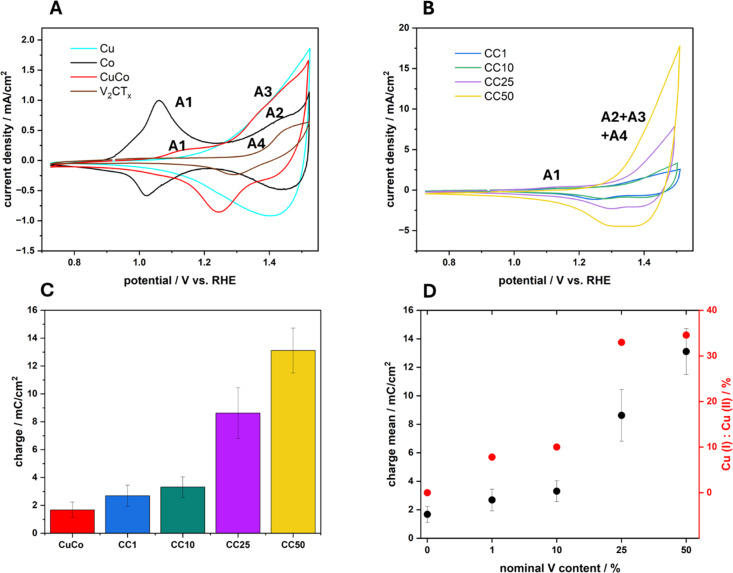
Cyclic voltammograms of (A) the pure Cu, Co, CuCo and V_2_CT_*x*_ materials and (B) the chemically functionalized hybrid materials CC1, CC10, CC25 and CC50, recorded at a scan rate of 40 mV in 1 M NaOH. (C) Charges for anodic sweeps and (D) charge of anodic sweep *vs.* Cu(i)/Cu(ii) % from XPS data.

The electrocatalytic activity of the four composite materials with different amounts of V_2_CT_*x*_ as well as the pure V_2_CT_*x*_, Cu, Co and CuCo was investigated toward OER. First, cyclic voltammetry was conducted at a scan rate of 40 mV s^−1^ to investigate the redox transitions of each material (Fig. 6A and B).

The pure Co catalyst exhibits an oxidation peak at 1.06 V *vs.* RHE which can be assigned to the redox transition of Co(ii) to Co(iii) (A1).^2^ For the same material, a second oxidation peak can be observed around 1.43 V *vs.* RHE, indicating the transition of Co(iii) to Co(iv) (A2).^2^ While the pure Cu material exhibits a reversible oxidation peak at around 1.40 V *vs.* RHE, indicating the transition of Cu(i) to Cu(ii) (A3). The pure V_2_CT_*x*_ has an oxidation peak at 1.43 V *vs.* RHE which can be attributed to a V(iv) to V(v) redox transition (A4) according to Pourbaix diagrams.^34^ For the mixed metal composite CuCo, an oxidation peak at 1.08 V *vs.* RHE can be attributed to the oxidation of Co(ii) to Co(iii) (A1), which is slightly shifted to higher potentials and significantly decreases in charge when compared to the pure Co material. The second peak for the mixed CuCo is observed at 1.44 V *vs.* RHE which may include the simultaneous transition of Co(iii) to Co(iv) and Cu(i) to Cu(ii) (A2 and A3).

In relation to the CC1-50 materials, Fig. 6B, the anodic peak (A1) for the Co(ii) to Co(iii) redox transition occurs at similar potentials however, lower current densities (Fig. S9†) compared to the mixed CuCo (Fig. 6A). Interestingly, an increase in the redox capacitance of the grouped A2–A4 peak of about 1.44 V *vs.* RHE with increasing V_2_CT_*x*_ in the sample is observed (Fig. 6C). However, the relative amount of V_2_CT_*x*_ in the composite samples is less than in the pure V_2_CT_*x*_ hence, the increase in the redox capacitance for the CC materials maybe be due to various phenomena. The first reason may be due to the increase in the vanadium content at the surface of the CC materials, which promotes electron transfer in the Cu and Co redox species due to enhanced conductivity, Fig. 5B.^35^ The second explanation for the increased redox capacitance could be related to the increased Co species at the surface which could cause the increase in charge due to Co(iii) to Co(iv) transition, Fig. 6B. Finally, the third plausible reason for the increased redox charge could be due to the higher amount of Cu(i) species with increasing V_2_CT_*x*_ content, Fig. 6D. The peak at 1.44 V *vs.* RHE, as previously mentioned, can also be assigned to the Cu(i) to Cu(ii) redox transition and if more Cu(i) species are present on the surface of a material before this redox transition the resulting redox peak will have a greater charge due to the formation of Cu(ii).

**Fig. 7:**
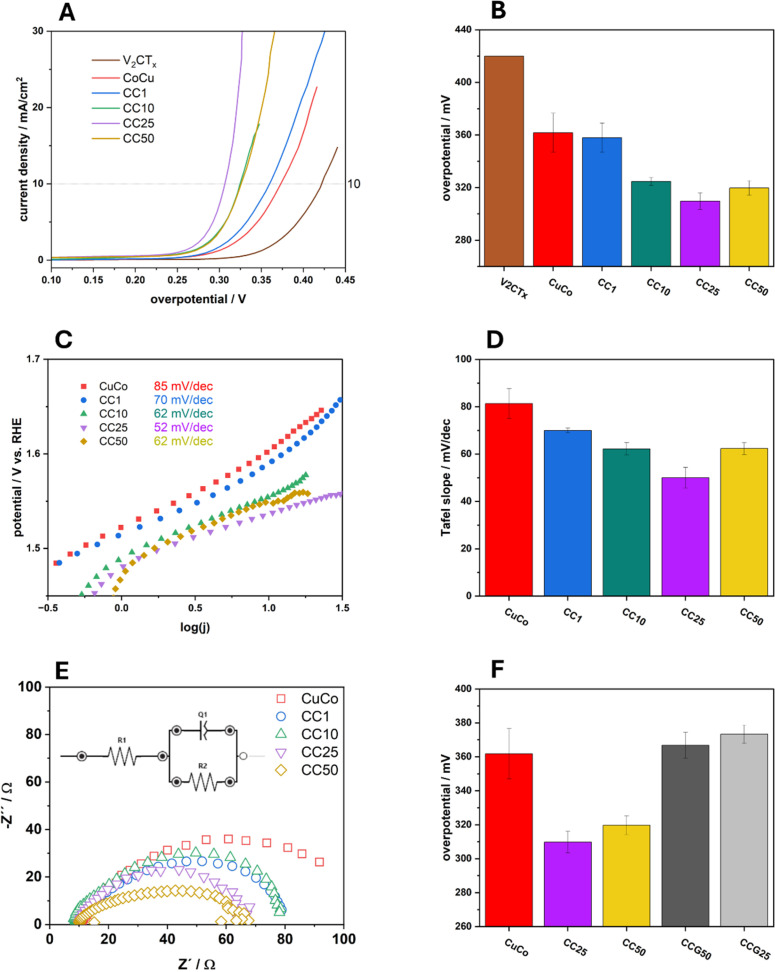
(A) LSV curves of the prepared materials in 1 M NaOH (B) corresponding overpotentials at 10 mA cm^−2^ (C) Tafel slope plots (D) corresponding Tafel slopes (E) Nyquist plots in OER region and (F) comparison to graphene composites.

Typical linear sweep voltammograms of the pure and composite catalysts in 1 M NaOH can be observed in Fig. 7A and overpotential values at 10 mA cm^−2^ (*η*_10_) of the average of three independent electrodes with standard deviations for each catalyst are presented in Fig. 7B. The pure V_2_CT_*x*_ catalyst exhibits an average overpotential at 10 mA cm^−2^ of 420 mV while the overpotential at the same current density for the pure CuCo is 360 mV. The chemically functionalized materials all show lower *η*_10_ values compared to the pure material, which shows their increased electrocatalytic activity regarding the OER. For the 1%, 10% and 25% CC materials, the *η*_10_ values become lower, indicating that subsequent additions of V_2_CT_*x*_ to 25% improves the OER. With 50% V_2_CT_*x*_, the OER performance slightly decreases, however the performance is equal to that of the 10% CC material.

The Tafel slope values for the pure and composite materials were also calculated and are presented in Fig. 7C. The same trend for the Tafel slopes values, Fig. 7D, can be observed as in Fig. 7B for the overpotentials at 10 mA cm^−2^. The Tafel slope value for the pure CuCo materials is 85 mV dec^−1^ which decreases to 70 mV dec^−1^ for the CC1, then to 62 mV dec^−1^ for CC10 and, finally 52 mV dec^−1^ for the CC25. This decrease in Tafel slope value shows that the chemical functionalization of the V_2_CT_*x*_ to the pure CuCo materials allows the OER to proceed at a faster rate than the pure CuCo. For the CC 50 material, the Tafel slope increases to 62 mV dec^−1^ which indicates the rate of the OER in the measured region is slowing down compared to CC25. This may indicate that there is an optimum amount of V_2_CT_*x*_ which is beneficial to the system for improving the OER, especially since the same trend is observed for the overpotential at 10 mA cm^−2^ in Fig. 5B.

The Nyquist plots in Fig. 7E and Table S1† show the charge transfer characteristics of the pure and composite materials taken at 1.6 V *vs.* RHE during OER. It is evident that the CC25 and CC50 have similar charge transfer resistances, although the CC50 has a higher double layer capacitance due to significantly higher MXene content. The CC10 and CC1 exhibit roughly the same properties; however, exhibit reduced charge transfer properties compared to the CC25 and CC50. The pure CuCo exhibits the most sluggish charge transfer compared to MXene supported catalysts hence, showing that the V_2_CT_*x*_ (MXene) plays a major role in increasing the charge transfer properties of the pure CuCo during the OER.

In order to further validate that the enhanced OER performance of the V_2_CT_*x*_ MXene-containing composites is not only due to increased surface area or charge transfer properties, graphene was used as an alternative to the V_2_CT_*x*_ in the synthesis process. Two CuCo/graphene-based (CCG) composites were prepared by the same hydrothermal route containing 25 and 50% graphene to compare to the CC25 and CC50 to determine the overpotential at which these two graphene composites reach 10 mA cm^−2^, Fig. 9. It is evident from Fig. 9 that the graphene composites exhibit similar or higher overpotential values at 10 mA cm^−2^ when compared to the pure CuCo material and the V_2_CT_*x*_ MXene composites clearly outperform their graphene counterpart composites.

**Fig. 8:**
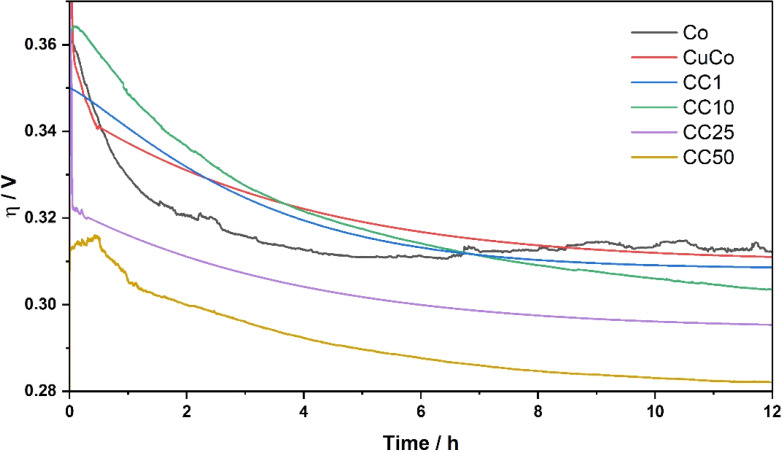
Stability performance of the pure and CC materials at 10 mA cm^−2^ over 12 hours.

Another important parameter for water splitting applications is the stability of the catalysts under operation for extended periods of time. The stability tests for the pure Co, pure CuCo and CC materials in this study were carried out at a current density of 10 mA cm^−2^ for 12 hours, Fig. 8.

**Fig. 9:**
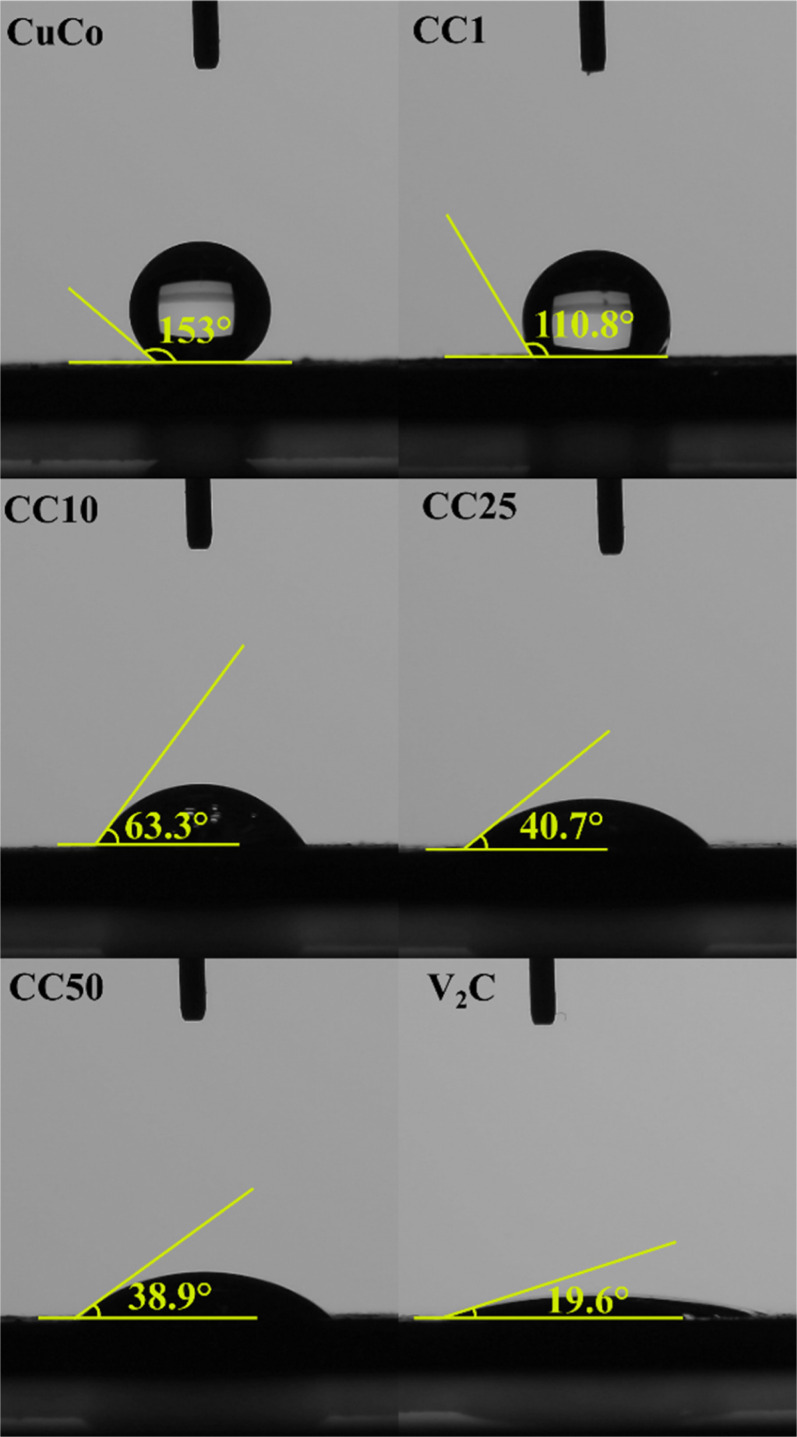
Contact angle measurements of the pure CuCo, V_2_CT_*x*_ and CC materials.

All the pure and composite material activities at the start of the stability test are improved within the first 1–4 hours when 10 mA cm^−2^ is applied. The pure Co catalyst starts to slightly decline around 6 hours. Interestingly, for all the materials that contain Cu, the stability is constant after the 6 hour mark, or the activity improves. At 12 hours of operation, the activity of the CC50 shows the best OER activity of ∼285 mV at 10 mA cm^−2^, which is retained after 24 hours of operation, Fig. S10.† It is evident from the stability tests that the addition of the Cu to the Co stabilizes the CuCo compared to the Co during the stability test while the addition of the V_2_CT_*x*_ has a significant effect on the overall activity over prolonged times. The V_2_CT_*x*_ is clearly advantageous in the composite as a pre-catalyst as the CC25/50 composites in this study as it out-performs similar materials made from Co, Cu and V in the literature, Table S2.†

### 3.3. Discussion of improved activity for the MXene composite materials

To elucidate the reasoning behind the improvement of the composite catalysts, the effect of the MXene on the hydrophilicity of the surface was investigated alongside post-mortem XPS to gain insight to the oxidation states and ICP-OES to determine dissolution rates of the Co, Cu and V.

First, the hydrophilic nature of the pure and composites were determined by contact angle measurements, Fig. 9. This is because hydrophilic materials are advantageous in water splitting applications, such as the OER, due to the increased wettability of the catalyst surface when in contact with the aqueous electrolyte. V_2_CT_*x*_ when synthesized by the *in situ* method, which was used in this study, is known to yield hydrophilic materials in nature due to their OH surface termination sites.^36,37^

The contact angles of the pure CuCo and composite materials decreased from 153° to 38.9° in proportion to the increasing amount of V_2_CT_*x*_, while the pure V_2_CT_*x*_ exhibited the lowest contact angle of 19.6°. The addition of 1% of the V_2_CT_*x*_ lowered the contact angle in comparison to the pure CuCo materials, however, it was insufficient to change the hydrophobicity nature of the catalyst surface, as the angles were higher than 90°.^38^ For additions of 10% or more of V_2_CT_*x*_, the angles were less than 90°, confirming the hydrophilic behavior of the catalyst surface, which show that the MXene has a role in making the composites with 10% V_2_CT_*x*_ or more hydrophilic in nature, allowing the catalytic surface better accessibility to the aqueous electrolyte.

**Fig. 10:**
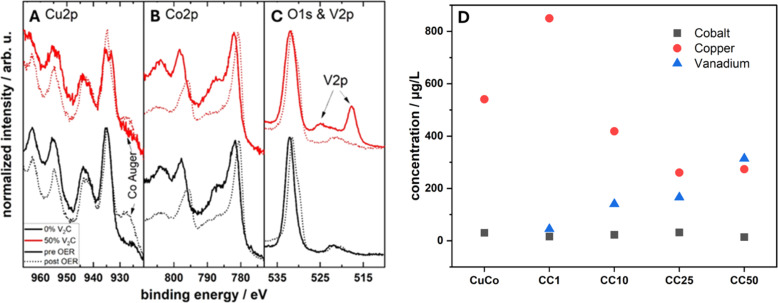
XPS post-mortem analysis for the pure CuCo and CC50 catalysts at (A) Cu 2p (B) Co 2p (C) O 1s and V 2p core levels and (D) ICP-OES measurements of the electrolyte used after OER stability tests.

Additionally, post-mortem XPS analysis was conducted to investigate any changes in the chemical states of the pure CuCo and the CC50 material (best performing composite due to the lowest *η* exhibited after 12 hours of stability test, Fig. 8), Fig. 10A–C. The Cu 2p region shows that the Cu oxide in the pure CuCo remains unchanged after the OER while the reduced Cu species in the CC50 has oxidized to Cu(ii), Fig. 10A. For both the pure CuCo and the CC50, the Co species is further oxidized after the OER, as evident from Fig. 10B. Interestingly, the vanadium at the surface of the CC50 is no longer detected, Fig. 10C, and the Co/Cu ratio has significantly increased compared to before OER. However, the Co/Cu ratio for the CC50 is lower than that of the pure CuCo which may indicate that copper leaches out of the pure CuCo at a faster rate during the OER. The lower leaching rate of the Cu/higher amount of the Cu on the surface of the CC50 may be influenced by the V_2_CT_*x*_ in the pre-catalyst, which itself might be preferentially leached during OER, as there is no V 2p signal after OER from the XPS, Fig. 10C.

To investigate the leaching effect of the Co, Cu and V during the OER, Inductively Coupled Plasma Optical Emission Spectroscopy (ICP-OES) was carried out on the pure CuCo and the composite materials after stability tests, Fig. 10D. From the ICP-OES results, it is clear that for all of the materials, Co leaching from the electrode into the electrolyte is minimum compared to the Cu leaching. However, interestingly, the amount of the V_2_CT_*x*_ does have a significant effect on the amount of Cu leached during the OER. For the composite materials, the material with only 1% V_2_CT_*x*_, has the highest amount of Cu leached during the OER, and the Cu leaching decreases with increasing V_2_CT_*x*_. Furthermore, for the CC50, the materials with the highest amount of V_2_CT_*x*_, also exhibits the highest amount of V_2_CT_*x*_ leaching. This result confirms that the V_2_CT_*x*_ is preferentially leached instead of the Cu postulated from the post XPS analysis. Hence, the increased performance of the CC50 during the stability tests compared to the other composites may be a result of more Cu remaining in the CuCo materials in the case of the CuCo V_2_CT_*x*_ composites.

## 4. Conclusions

A series of CuCo/V_2_CT_*x*_ based composites were prepared by a hydrothermal synthesis method with the V_2_CT_*x*_ component ranging from 0 to 50%. The pure and composite materials were identified to be hydroxide carbonate materials by XRD. The XRD showed that for the composite materials, the (002) reflection of the V_2_CT_*x*_ shifted to show smaller interlayer spacings observed for multi-layered V_2_CT_*x*_, suggesting restacking during synthesis. The STXM measurements confirm that there are no changes in the vanadium oxidation state in the bulk of the materials when comparing the pure V_2_CT_*x*_ and the CC50 after synthesis, showing that no bulk oxidation of the V_2_CT_*x*_ occurs during the hydrothermal synthesis. However, surface oxidation of the vanadium in the composite materials is observed from the XPS analysis compared to the pure V_2_CT_*x*_. XPS further revealed that the Cu(ii) oxidation state was reduced with increasing MXene added to the composite and the Co component compared to the Cu increased at the surface.

Extensive OER performance analysis was carried out on the pure and composite samples and the conclusion was that the CC25-50 materials are the most active for the OER in this study. This increase in OER activity may be related to the various changes in CuCo properties due to the V_2_CT_*x*_ in the pre-catalyst including the increase in hydrophilicity of the catalyst surface and increase in charge transfer properties. Additionally, the initial increased activity from the LSVs could be attributed to the interplay of the higher at% of the Co and the reduced Cu oxidation state leading to an ideal pre-catalyst structure on the surface as determined by XPS.

The OER activity of the same CuCo materials with graphene were investigated as well and showed that the enhancement is not just due to the high surface area or conductivity of the MXene as the graphene composites were equal to or exhibited decreased activity for the OER when compared to the pure CuCo material. Furthermore, all the CC V_2_CT_*x*_ composites became more active for the OER during the stability tests at 10 mA cm^−2^ which is attributed to the preferential leaching of the V_2_CT_*x*_ over the Cu. Hence, overall, this allowed for a higher concentration of the CuCo active site to remain on the electrode surface thus resulting in higher OER performances for the composites with higher V_2_CT_*x*_*i.e.* the CC50.

## Data availability

Some of the data supporting this article have been included as part of the ESI.† Additionally, the raw data files for this study can be made available by contacting the corresponding author.

## Author contributions

Bastian Schmiedecke: investigation, data curation, writing – original draft, writing – review & editing. Bing Wu: investigation, writing – review & editing. Thorsten Schultz: investigation, writing – review & editing. Aline Alencar Emerenciano: investigation, writing – review & editing. Namrata Sharma: investigation, writing – review & editing. Danielle A. Douglas-Henry: investigation, writing – review & editing. Apostolos Koutsioukis: investigation, writing – review & editing. Mehmet Turan Görüryılmaz: investigation, writing – review & editing. Valeria Nicolosi: supervision, writing – review & editing. Tristan Petit: supervision, writing – review & editing. Norbert Koch: supervision, writing – review & editing. Zdenek Sofer: supervision, writing – review & editing. Michelle P. Browne: writing – original draft, writing – review & editing, data curation, supervision, investigation, funding acquisition, conceptualization.

## Conflicts of interest

There are no conflicts to declare.

## Supplementary Material

TA-012-D4TA02700K-s001
