# Supplementary material for: Enhancing the oxygen evolution reaction activity of CuCo based hydroxides with V2CTx MXene
Source: J Mater Chem A Mater. 2024 Aug 2;12(36):24248–59. doi: 10.1039/d4ta02700k (PMC11325309; doi:10.1039/d4ta02700k)
Supplement: TA-012-D4TA02700K-s001 [file TA-012-D4TA02700K-s001.pdf]

Supplementary Information (SI) for Journal of Materials Chemistry A.
This journal is © The Royal Society of Chemistry 2024

Supporting Information

Enhancing the Oxygen Evolution Reaction activity of CuCo based Hydroxides with V_2CT_x MXene

Bastian Schmiedecke[1], Bing Wu[2], *Thorsten Schultz*[3,4], *Aline Alencar Emerenciano*[1], *Namrata Sharma*[5], *Danielle A. Douglas-Henry*[6], *Apostolos Koutsioukis*[6], *Mehmet Turan Görüryılmaz*[1], *Valeria Nicolosi*[6], *Tristan Petit*[5], *Norbert Koch*[3,4], *Zdenek Sofer*[2] *and Michelle P. Browne*[1*]

1. Helmholtz Young Investigator Group Electrocatalysis: Synthesis to Devices, Helmholtz-Zentrum Berlin für Materialien und Energie GmbH, Albert-Einstein-Str. 15, 12489 Berlin, Germany.
2. Department of Inorganic Chemistry, University of Chemistry and Technology, Prague, Technická 5, 166 28 Prague 6, Czech Republic.
3. Helmholtz-Zentrum Berlin für Materialien und Energie GmbH, Berlin, 14109, Germany.
4. Institut für Physik & IRIS Adlershof, Humboldt-Universität zu Berlin, Berlin, 12489, Germany.
5. Helmholtz Young Investigator group Nanoscale Solid−Liquid Interfaces, Helmholtz-Zentrum Berlin für Materialien und Energie GmbH, Albert-Einstein-Str. 15, 12489 Berlin, Germany
6. School of Chemistry, CRANN and AMBER Research Centres, Trinity College Dublin, College Green, Dublin D02 PN40, Ireland.

***Email:** Michelle.browne@helmholtz-berlin.de

Materials and Methods continued

Graphene and graphene composite synthesis

To prepare a typical graphene dispersion, 180 mg of graphite was dispersed in 180 ml of isopropyl alcohol (IPA) (starting concentration of graphite in IPA: 1 mg ml^{-1}) using a 1.3 hour sonication process at 60 % amplitude, with a pulsation pattern of 6 seconds on and 2 seconds off to avoid damage to the processor and reduce solvent heating, and, thus, evaporation. The beaker was connected to a cooling system that allowed for cold water (under 5 °C) to flow around the dispersion during sonication. The resulting dispersion was centrifuged for 90 minutes at 500 rpm. After centrifugation, the upper 45 ml of the dispersion was retained for use. The final concentration of graphene nanosheets in IPA were 0.5 mg ml^{-1}.

CuCo@graphene (CCG) was synthesized through a urea-assisted hydrothermal method. In a typical synthesis process 5 mmol urea, 1 mmol $Cu(CH_3COO)_2$• H_2O and 2 mmol $Co(CH_3COO)_2$• 4 H_2O were added to a graphene suspension, and it was stirred for 30 minutes to fully dissolve all the compounds. The solution was transferred into a Teflon-lined stainless-steel autoclave and kept at 120 °C for 6 h for hydrothermal treatment. After cooling down to room temperature, the precipitate was collected by centrifugation at 5000 rpm for 10 min, and then repeatedly washed with deionized water (3 x) and ethanol (3 x). The sediment was dried overnight at 60 °C for 10 h. The materials were labelled CCG50 and CCG25 for 50 % and 25 % graphene content, respectively.

Material characterizations

The phase analysis of the samples was performed at room temperature (RT) by powder X-ray diffraction (XRD) utilizing a Bruker D8 ADVANCE powder diffractometer with Cu-K_{α} radiation of 40 kV and a beam current of 40 mA (λ(Cu-$K_{\alpha1}$) = 0.1541 nm and λ(Cu-$K_{\alpha2}$) = 0.1544 nm). Diffraction patterns were collected between 3.5° and 80° applying a step size of 0.015°. The morphologies of the as-prepared samples were observed using a Karl Zeiss MERLIN scanning electron microscope (SEM) using a 0.1–30 keV field emission gun. X-ray photoelectron spectroscopy (XPS) measurements were conducted using a JEOL JPS-9030 setup with a base pressure of 2E-9 mbar. The powders were evenly distributed on carbon tape for the measurements. A non-monochromated Al source with 300 W power was used for excitation and a hemispherical analyzer with pass energy of 50 eV (surveys) and 20 eV (narrow scans) was used to detect the emitted photoelectrons. The analyzer binding energy scale was calibrated by measuring sputter cleaned gold and copper foils just before the measurements and setting the Au4f_7/2 peak to 84.00 eV and the Cu2p_3/2 peak to 932.62 eV. Since the samples exhibited charging, the C-C component of the carbon tape was set to 285.0 eV for comparison. CasaXPS was used to fit the spectra, employing Tougaard (Cu2p) and Shirley (all other core levels) backgrounds and Voigt-functions. X-ray Absorption Near Edge Structure (XANES) spectra was acquired using Scanning Transmission X-Ray Microscopy (STXM) at the BESSY-II electron storage ring. The measurements were performed at the MAXYMUS end station. The x-ray beam was focused with a zone plate and an order selective aperture on the transmissive sample. The samples were measured ex-situ under vacuum (~10^{-6} mbar). Scanning Transmission Electron Microscopy (STEM) was performed using a FEI Titan 80–300 Thermo

Fisher Scientific, fitted with a Schottky field emission gun set to an operating voltage of 300 kV. Elemental composition was determined by Energy Dispersive X-Ray Spectroscopy (EDS) using a Bruker XFlash 6–30 EDS detector. Au grids TEM grids were used to allow for the true Cu signal of the materials to be detected. Contact angle measurements were carried using an Ossila Contact Angle Goniometer, in which a deionised water droplet (5 µl) was used to characterize the wettability of the catalysts. The Inductively coupled plasma optical emission spectroscopy (ICP-OES) measurements were carried out with a iCAP 7400 DV from ThermoFisher in axial measurement mode.

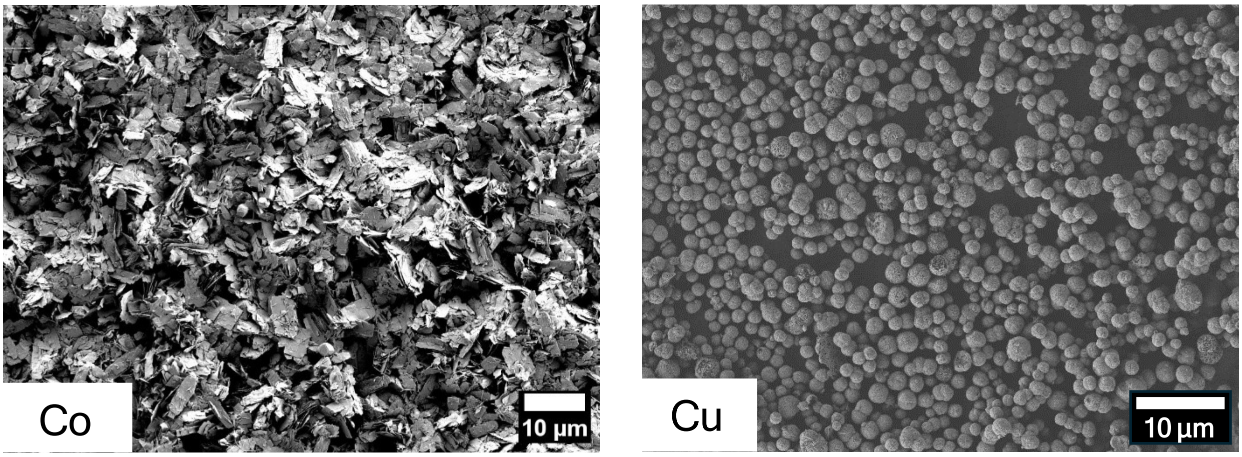

Figure S1. SEM images of pure Co and pure Cu.

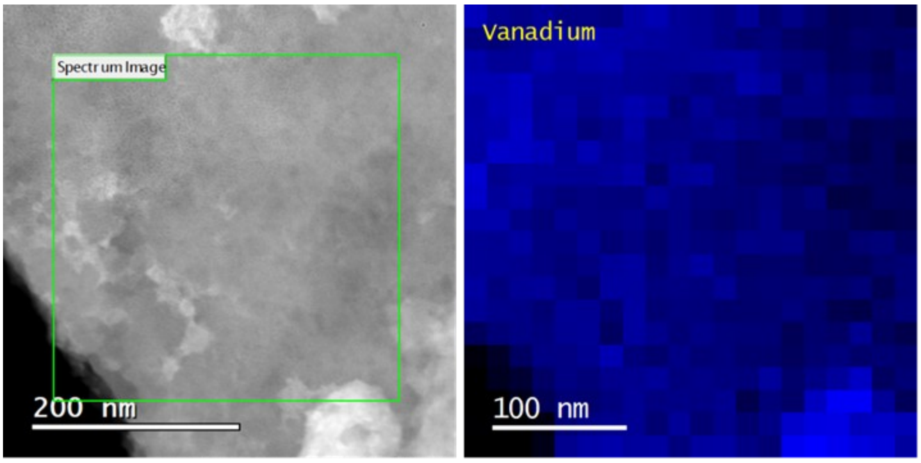

Figure S2. EDS map of V_2CT_x.

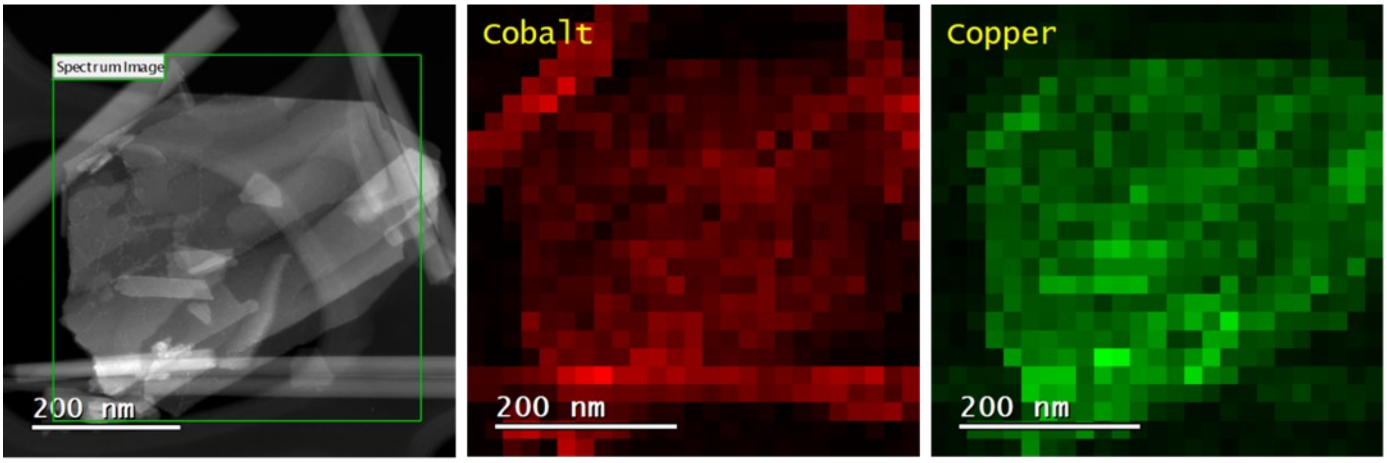

Figure S3. EDS map of pure CuCo.

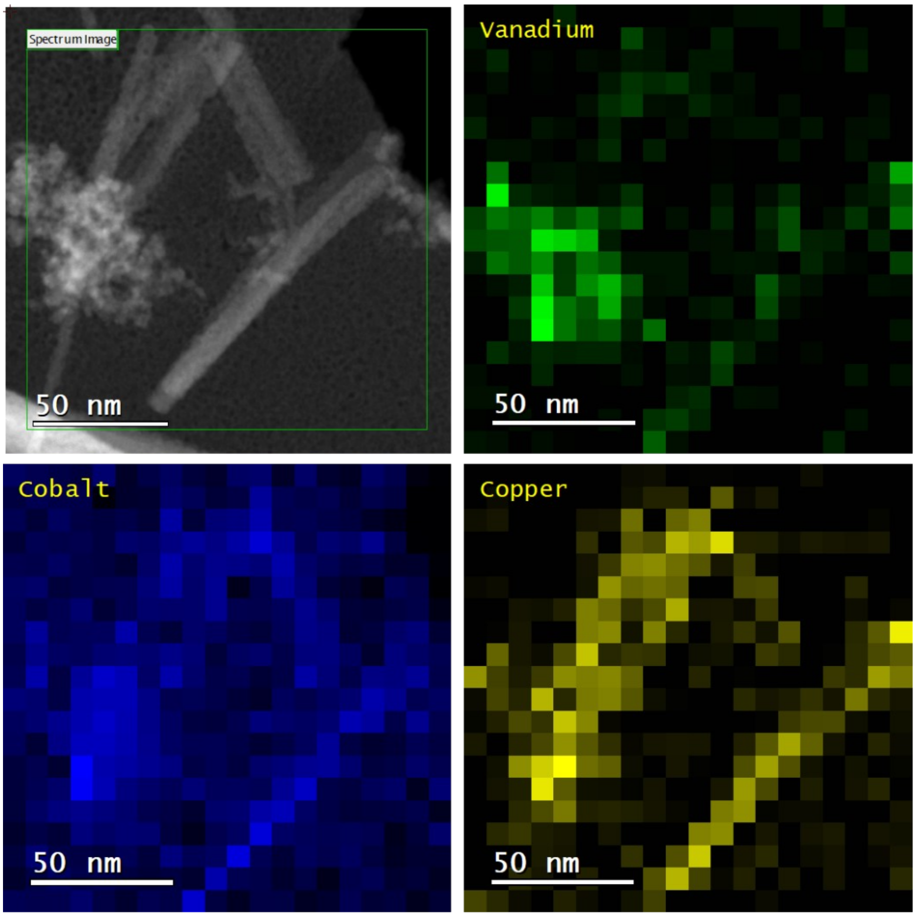

Figure S4. EDS map of CC1.

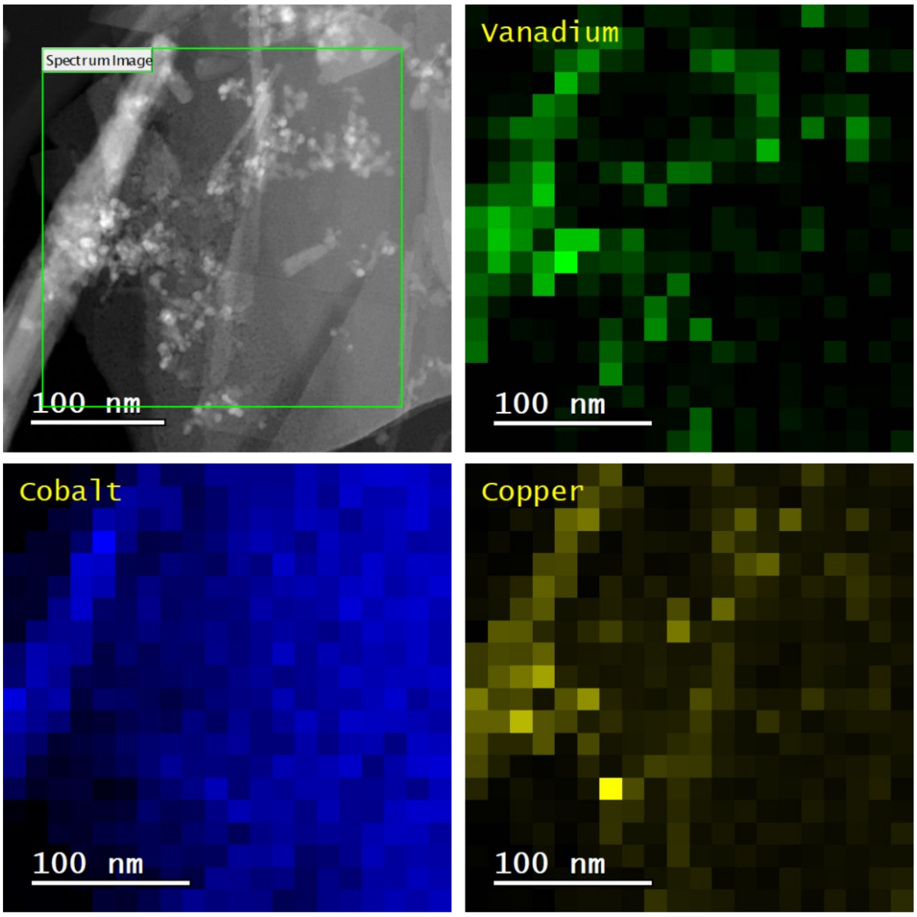

Figure S5. EDS map of CC10.

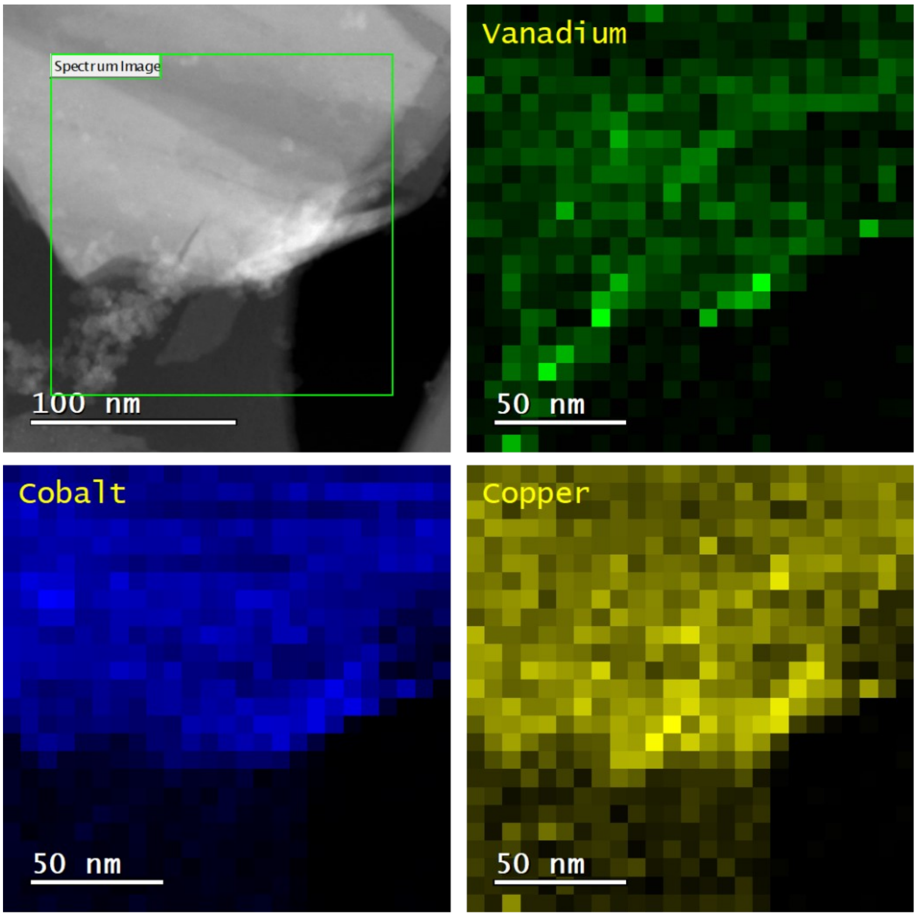

Figure S6. EDS map of CC25.

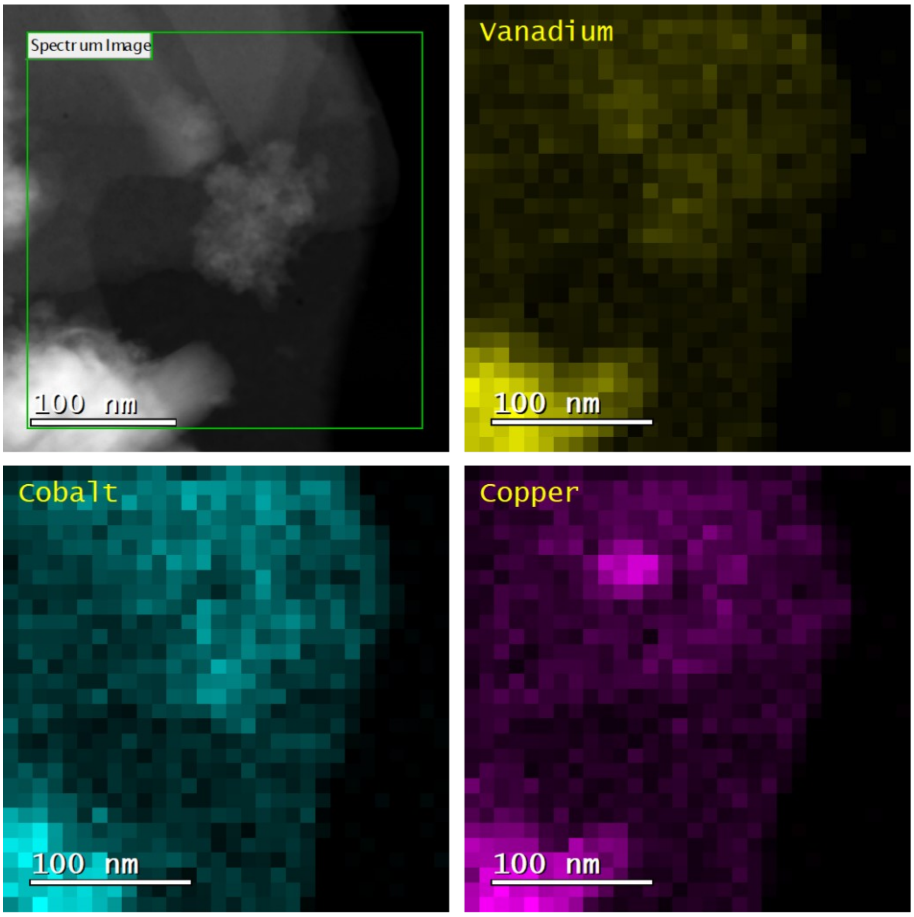

Figure S7. EDS map of CC50.

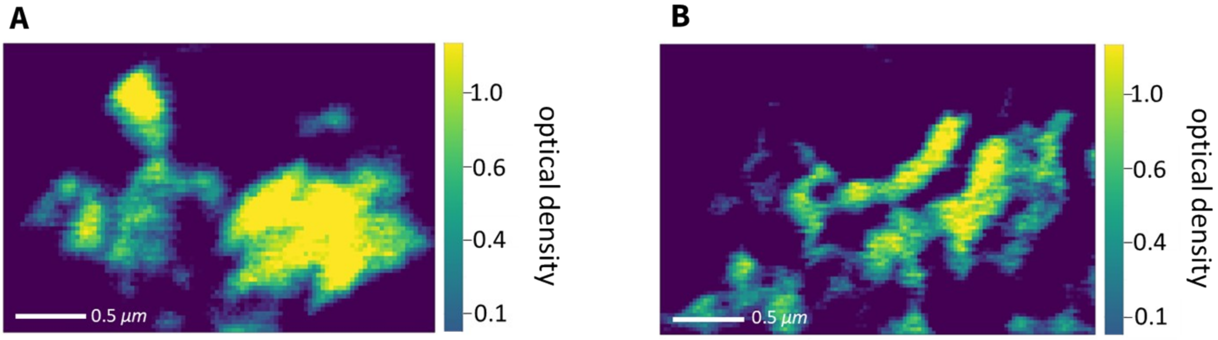

Figure S8. Transmission image **A.** at maximum absorption of V-L_3 edge at 517 eV of pristine V_2CT_X, and **B.** of CC50. NOTE: The XANES spectra in Figure 3C in the main paper is an averaged spectra over these images. Lower optical density represents the background and correspondingly increasing contrast indicates the thickness of the sample.

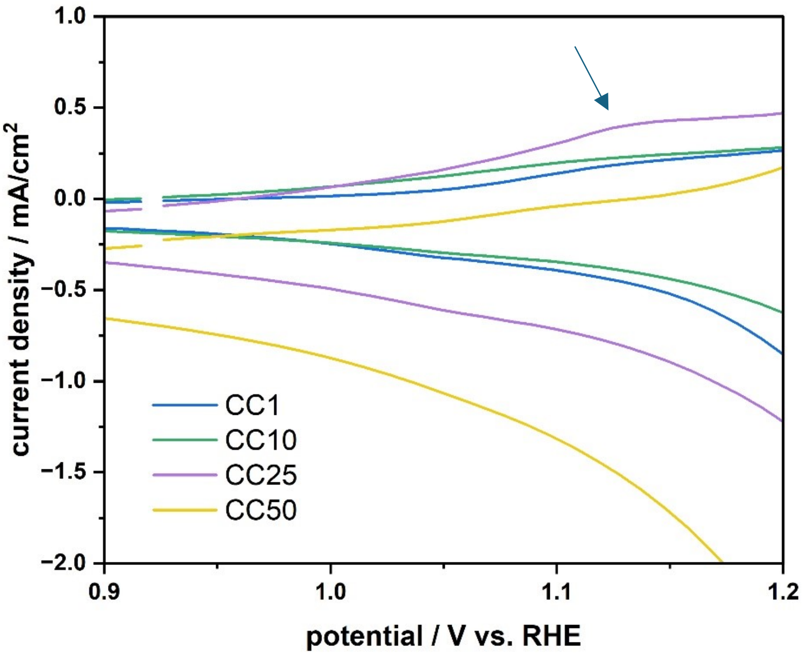

Figure S9. CV of the CC composite materials highlighting the A1 redox peak.

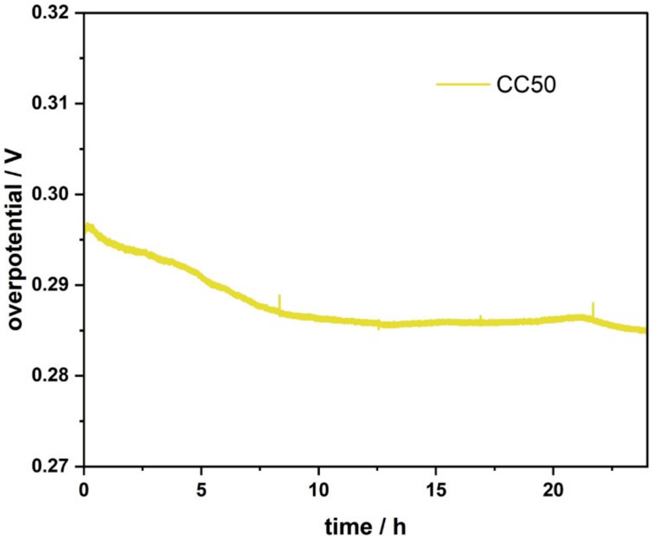

Figure S10. Stability test of the CC50 for 24 hours.

Table S1. Fitted values of the charge transfer resistance during OER.

**Material**	**R_{ct} [Ω]**
CuCo	102.3
CC1	74.37
CC10	76.54
CC25	58.30
CC50	53.81

Table S2. Literature comparison of Cu/Co/V based materials.

Catalyst	Overpotential at 10 mA cm^{-2} (mV)	Stability	Preparation method	Reference
$Cu_xCo_{3-x}O_4$@C	385	5000 cycles CV	Hydrothermal	[1]
Co_3O_4@$Ti_3C_2T_x$	300	2000 cycles CV	Solvothermal	[2]
Co-LDH@ $Ti_3C_2T_x$	330	20 h galvanostatic	Solvothermal	[3]
CuCoO-NWs	320	50 h @ 1.5 V vs RHE	Hydrothermal	[4]
Cu-Co_3O_4 (Cu/Co = 1/8)	391	2 h @ 0.8 V vs Ag/AgCl	Solid-solid	[5]
NiV-LDH	318	25 h chronopotentiometry	Hydrothermal	[6]
$CoVO_x$ (3:1)	351	15 h	Hydrothermal	[7]
CuCu@V_2CT_X (50)(CC50)	285	24 h and improvement in activity	Hydrothermal	This work

References

1. W. Jin, J. Chen, Z. Wu and G. Maduraiveeran, *International Journal of Hydrogen Energy*, 2019, **44**, 11421-11430.
2. Y. Lu, D. Fan, Z. Chen, W. Xiao, C. Cao and X. Yang, *Science Bulletin*, 2020, **65**, 460-466.
3. M. Benchakar, T. Bilyk, C. Garnero, L. Loupias, C. Morais, J. Pacaud, C. Canaff, P. Chartier, S. Morisset, N. Guignard, V. Mauchamp, S. Célérier and A. Habrioux, *Advanced Materials Interfaces*, 2019, **6**, 1901328.
4. M. Kuang, P. Han, Q. Wang, J. Li and G. Zheng, *Advanced Functional Materials*, 2016, **26**, 8555-8561.
5. T. Grewe, X. Deng, C. Weidenthaler, F. Schüth and H. Tüysüz, *Chemistry of Materials*, 2013, **25**, 4926-4935.
6. K. Fan, H. Chen, Y. Ji, H. Huang, P. M. Claesson, Q. Daniel, B. Philippe, H. Rensmo, F. Li, Y. Luo and L. Sun, *Nature Communications*, 2016, **7**, 11981.
7. L. Liardet and X. Hu, *ACS Catalysis*, 2018, **8**, 644-650.
